# Dynamics of Whale Shark Occurrence at Their Fringe Oceanic Habitat

**DOI:** 10.1371/journal.pone.0102060

**Published:** 2014-07-16

**Authors:** Pedro Afonso, Niall McGinty, Miguel Machete

**Affiliations:** 1 IMAR - Institute of Marine Research. Department of Oceanography and Fisheries, University of the Azores, Horta, Portugal; 2 LARSyS - Laboratory of Robotics and Systems in Engineering and Science, Lisbon, Portugal; 3 MARICE - Marine Academic Research in Iceland. Department of Life and Health sciences, University of Iceland, Reykjavik, Iceland; Institut Maurice-Lamontagne, Canada

## Abstract

Studies have shown that the whale shark (*Rhincodon typus*), a vulnerable large filter feeder, seasonally aggregates at highly productive coastal sites and that individuals can perform large, trans-boundary migrations to reach these locations. Yet, the whereabouts of the whale shark when absent from these sites and the potential oceanographic and biological drivers involved in shaping their present and future habitat use, including that located at the fringes of their suitable oceanic habitat, are largely unknown. We analysed a 16-year (1998–2013) observer dataset from the pole-and-line tuna fishery across the Azores (mid-North Atlantic) and used GAM models to investigate the spatial and temporal patterns of whale shark occurrence in relation to oceanographic features. Across this period, the whale shark became a regular summer visitor to the archipelago after a sharp increase in sighting frequency seen in 2008. We found that SST helps predicting their occurrence in the region associated to the position of the seasonal 22°C isotherm, showing that the Azores are at a thermal boundary for this species and providing an explanation for the post 2007 increase. Within the region, whale shark detections were also higher in areas of increased bathymetric slope and closer to the seamounts, coinciding with higher chl-a biomass, a behaviour most probably associated to increased feeding opportunities. They also showed a tendency to be clustered around the southernmost island of Santa Maria. This study shows that the region integrates the oceanic habitat of adult whale shark and suggests that an increase in its relative importance for the Atlantic population might be expected in face of climate change.

## Introduction

The search for favourable local conditions across marine habitats is broadly considered to be the main evolutionary force driving large pelagic predators to migrate across the oceans. These conditions can be multiple across an individual's lifetime, such as a predator free environment for the survival of newly born offspring, the availability of prey or a temperature envelope within the physiological constraints of the animal. Because environmental conditions can change drastically, this dynamic provides the basis for the development of migratory behaviours. For example, some oceanic sharks are known to visit and depend on specific coastal habitats to pup or to feed as young [1]–[3]. An interesting consequence of this hypothesis is the importance of habitats lying at the geographic limits of a migratory species' habitat range, particularly given the current evidences of climate change and their effects on fish species ranges [4]. If conditions change in the longer-term, can such a fringe habitat become more important to a population of a migratory large pelagic predator?

The whale shark (*Rhincodon typus*, Smith 1828) is the largest living fish, inhabiting all tropical and warm temperate seas apart from the Mediterranean [5]. In contrast with the vast majority of other elasmobranchs, it has evolved a migratory lifestyle associated to a filter feeding ecology. Up until 1985 only 320 records of this species were in existence [6] and, in spite of the substantial research in the last two decades, especially in the Indo-Pacific, knowledge of the species' ecology, life history and fisheries is still relatively scarce [7]–[8]. The information available so far from several tracking studies shows that the whale shark aggregates seasonally at particular tropical, coastal sites, and that individuals can perform large, trans-boundary migrations in search for productive patches of plankton and nekton to feed upon [9]–[10]. Recent modelling work based on fisheries data showed that whale shark habitat suitability in the Indian Ocean is mainly correlated with spatial variation in sea surface temperature (SST) and that the species shows a strong preference for a restricted ocean surface temperature between 26.5 and 30°C [11]. Other studies have also shown that annual presence at aggregation sites can be highly influenced by interannual changes in SST [12] or climatic forcing [13]–[15]. However, the location, temporal stability and connectivity of oceanic habitats for the whale shark and the way in which these habitats can be affected by climate change are major conservation questions that remain to be answered [16].

**Figure 1:**
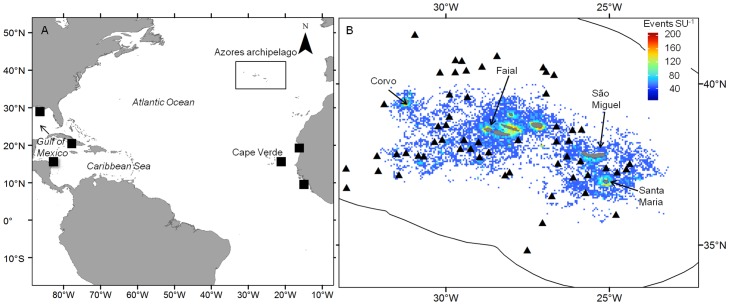
The Azores archipelago and the tuna fishing effort. (A) The location of the Azores archipelago in the North Atlantic. Also shown are documented locations in the North Atlantic where aggregations of whale sharks have been known to occur (black squares) [17]. (A) Position of the nine islands that constitute the Azores archipelago together with the distribution of effort in the cumulative number of fishing events SU-1 within the Azores EEZ (black line). Large seamounts are represented by black triangles. Also shown are the names of the islands mentioned in the text.

The large scale seasonal movements of whale sharks have been shown to be essentially longitudinal between different tropical or subtropical regions [8]. However, the whale shark can also occur in regions at higher latitudes such as the Azores archipelago, the northern limit of its geographic Atlantic distribution located at the boundary between the subtropical and warm temperate North Atlantic provinces (Figure 1). Whale sharks − local name ‘*pintados*’ − are known by Azorean fishermen to sometimes occur during the summer and associate with tuna, and have been used for a long time as an aid to locating and fishing the tuna schools. Whether this region is only sporadically visited by whale sharks or is part of their normal oceanic habitat is as yet unknown. In addition, a surge in whale shark occurrence was reported by fishermen to occur in the region during the last few years, this putative increase being the justification for the onset of a recent whale shark watching industry. These facts attribute the region as high interest due to its position at the fringes of the whale shark habitat and as a prime area to understand the potential impacts of climate change in the spatial ecology of large pelagic predators.

This is the first paper dealing with the occurrence of the whale shark in the central North Atlantic, and anywhere in their fringe habitats globally. We analyse a unique 16-year fisheries dataset to investigate the spatiotemporal patterns of occurrence in relation to some oceanographic features, and use this analysis to discuss 1) if there was an increase in visits of whale sharks to the Azores region, 2) if broadscale climatic forcing may have affected whale shark occurrence in this region across the years, and 3) which environmental cues may help explain their habitat use once they reach the region. Given the preference of the whale shark for high temperatures, their recognized seasonal occurrence in the region [16] and the predicted long-term shift in distribution towards the pole in the Atlantic [17], we ask whether changes in ocean characteristics in a climate change scenario, namely temperature and productivity regimes, will potentially alter the relative importance of this fringe habitat for the North Atlantic whale shark population.

## Materials and Methods

### Ethics statement

This study was performed according to national Portuguese laws for the use of vertebrates in research. All procedures followed the guidelines for the use of fishes in research of the American Fisheries Society. No animals were manipulated or sacrificed. All data were obtained during regular commercial fishery operations under the Azorean Fisheries Observer Program (POPA) jointly run by IMAR- UAz and the Regional Government of the Azores (SRRN/DRP).

### Sightings

The Azores archipelago consists of nine islands that are broadly grouped into the western, central and eastern island groups (Figure 1A) and is located along the mid-Atlantic ridge in the Atlantic Ocean (Figure 1b). We used the pole-and-line tuna fisheries database obtained from the observation programme for fisheries of the Azores (POPA) to look for whale shark sightings in the wider Azores region. The program was launched in 1998 and annually deploys observers in around 20 of the large tuna vessels which equates to approximately 50% of the tuna fleet. It operates with trained on-board observers who record georeferenced data on all tuna fishing activities and other scientifically relevant information including fishing effort, tuna catch, and sighting of different associated and non-associated species such as turtles, marine mammals and whale sharks. The tuna season typically extends from May to November during which boats actively seek for tuna and come into port every 2–8 days, depending on the amount of fish caught. The observers are permanently deployed and observations may occur whenever to boat is out of the harbour. A fishing event with a whale shark associated to tuna will always result in the sighting of the animal very close to the boat's deck (<15 m). The average speed of the tuna vessels when searching at daytime is approximately eight knots (∼15 km h^−1^), meaning that a vessel will cover over 1300 linear km over a week. The presence of a whale shark swimming at or very close to the surface resulting in a fishing event will be detected by an observer covering 360° from the boat's flybridge. Telemetric studies done elsewhere [18] all found that whale sharks are known to spend the majority (∼80%) of their daytime very close to the surface, even if this behaviour is frequently interspersed with deep dives or permanence at deeper layers of the epipelagic domain. Therefore, we can reasonably assume that the probability of daytime whale shark detections by observers should be high and unbiased by their nighttime behaviour.

Fishing effort is not evenly distributed across the Azores Economic Exclusive Zone (EEZ, ca. 1 million km^2^). No approach can guarantee the removal of all double sightings in a location without further verification from photographic databases, therefore we propose a filtering technique to minimize potential duplicate sightings of the same individuals within each surveyed month. A screening process was applied to the raw data for the study area, whereby the region was divided into a regular grid of 4 km×4 km sampling units (SU; n = 24642), which corresponds to the highest spatial resolution available for environmental (remotely sensed) data. These SUs were used to filter the raw data per month by aggregating all events within each SU for each year/month combination. A whale shark was present within a SU if there was at least one sighting made within a calendar month. Although this does not remove the possibility of multiple detections of the same individual entirely, the monthly resolution between successive detections is expected to remove the vast majority of these occurrences. Pseudo-absence generation follows the recommended guidelines outlined by [19] where a large number of pseudo-absences are randomly generated with respect to each presence with a ratio of (1∶100), which allows for a greater sampling of the background conditions. For each presence, one hundred random pseudo-absences were generated for that month (centred on the 15th of each month) by randomly resampling the SU grid with replacement. This procedure ensured that the temporal distribution of presences and pseudo-absences retain the same proportion across all months and years.

### Environmental variables

Satellite derived estimates of both sea surface temperature (SST) and chlorophyll (chl*-a*) were extracted for the area for the years 1998–2013. In order to cover this full period, the satellite data for both SST and chl-*a* for this period had to originate from different sources: AVHRR1 (1998–2009) and MODIS2 nightime SST (2002–2013) for SST, and Globcolour database (1998–2013) for chl-a. The merging of both SST datasets required a regression of overlapping values in order to produce a full length dataset for each variable (Table 1). The North Atlantic Oscillation (NAO) index is a basin-scale alternation of atmospheric mass between the Subtropical and the Arctic Atlantic [19]. The index used here is the station based monthly index obtained from the NCAR database (http://www.cgd.ucar.edu/cas/jhurrell/indices. html). Bathymetry was derived from the General Bathymetric Charts of the Oceans (GeBCO) dataset [20], which is a global topographic relief model with a resolution of 0.086^o^ from which the average slope (in angular degrees) within each SU^−1^ was derived. The distance from the shore (m) was calculated for each sampled SU by using the distance from the SU midpoint to the nearest island shoreline. The level of fishing effort was also incorporated by using the frequency of fishing events to have occurred within each SU across all years. The sum of the fishing events within each month (events SU month^−1^) is used to verify if returning to the same area would have an effect on the probability of whale shark detection rates.

**Table 1:** Predictor variables used for GAM modelling of whale shark detections in the Azores EEZ.

Variables	Resolution		Information
	Spatial	Temporal	
SST (°C)	4 km	monthly (1998–2013)	The SST derived from AVHRR *The Advanced Very High Resolution Radiometer*; http://nsidc.org/data/avhrr/(1998-2009) and MODIS *Moderate Resolution Imaging Spectroradiometer* nighttime SST (2002–2013). Linear regression between datasets for overlapping periods: r^2^ = 0.87, slope = 0.97, intercept = 2.98.
chl-*a* (mg m^−3^)	4 km	monthly (2002–2013)	Chlorophyll is derived from the Globcolour observation program that provides merged ocean colour data from 4 satellite sources on a 4×4 km spatial grid. Data was downloaded from http://www.globcolour.info/
Effort (dimensionless)	4 km	(1998–2013)	The cumulative number of events per month within each SU. This gives an index of the fishing frequency for each SU.
Depth (m)	4 km	NA	The depth of the underlying bathymetry in meters derived from the GEBCO dataset.
Slope (degrees)	4 km	NA	The average slope in degrees of the underlying bathymetry (GEBCO) for each 4 km×4 km SU.
Distance to shore (m)	0 to 600 km	NA	The distance (m) of the monthly fishing event (marked as the centroid of each SU) to the nearest shoreline.
Distance to seamount (m)	0 to 300 km	NA	The distance (m) of the monthly fishing event (marked as the centroid of each SU) to the nearest seamount.
NAO (dimensionless)	NA	monthly	The values of the NAO monthly index based on the principal component (PC)-based indices of Empirical Orthogonal Function (EOF) of SLP anomalies over the Atlantic sector 20°–80°N, 90°W–40°E. These indices are used to measure the NAO throughout the year, tracking the seasonal movements of the Icelandic Low and the Azores High.

### Model selection

A model was developed to test which environmental variables might explain the probability of whale shark sightings across the Azores EEZ within the 16 year period of study. Monthly whale shark sightings (presence/absence) were set as the response variable and the spatial and non-spatial environmental and climatic variables outlined in Table 1 were used as predictors. Pearson correlations were first used to examine potential co-linearity between predictor variables. Highly correlated variables (r>0.7) were not included within the same models. Exploratory biplots revealed that the relationship between the response variable and several of the predictor variables were non-linear so a binomial generalized additive model (GAM) was chosen to model the whale shark data. GAM are regression models where smoothing splines are used instead of linear coefficients for covariates. The estimated degrees of freedom (edf) for each of the variables were limited to ≤3. This allowed for either linear, curvilinear or sigmoid relationships between whale shark presence/absences and the explanatory variables, which represent a reasonable range of relationships that could be expected and interpreted. Presences and absences across all years are initially split and are randomised separately to ensure an adequate number of presences in both datasets. A pseudo-random algorithm is implemented to randomly assign each presence and absence into the training and testing dataset in a ratio of 75∶25 respectfully. The model derived from the training GAM will be used to evaluate the predictive capabilities of the model using the receiver operating characteristic curve (ROC – e.g., [21]) on the test dataset. The ROC is used to determine the accuracy of the model by evaluating the models capability of discriminating between sites where a species is present versus sites where a species is absent with values approaching 1 indicating a greater ability for the model to discriminate between presences and absences. A stepwise procedure was used to select the most parsimonious model using the Akaike's Information criterion (AIC) function dropterms. There are concerns associated with stepwise procedures due to the potential of introducing variable bias and model overfitting, and several alternatives have been suggested to help counteract this problem [22]. We addressed this issue by following the recommended procedure outlined by [23] in which a double penalty approach is used together with a maximum likelihood (ML) estimator of the regression splines. By adding the extra penalty to the functions in the null space of the model, a variable that does not contribute significantly to the model can be completely removed and is a robust and powerful way of removing variable selection bias introduced through stepwise procedures [24]. The partial deviance explained (%) is then calculated for all variables present in the final model. All statistical analysis and model development were performed using packages (gam; *mgcv*) developed for the R programming environment [25].

## Results

**Figure 2:**
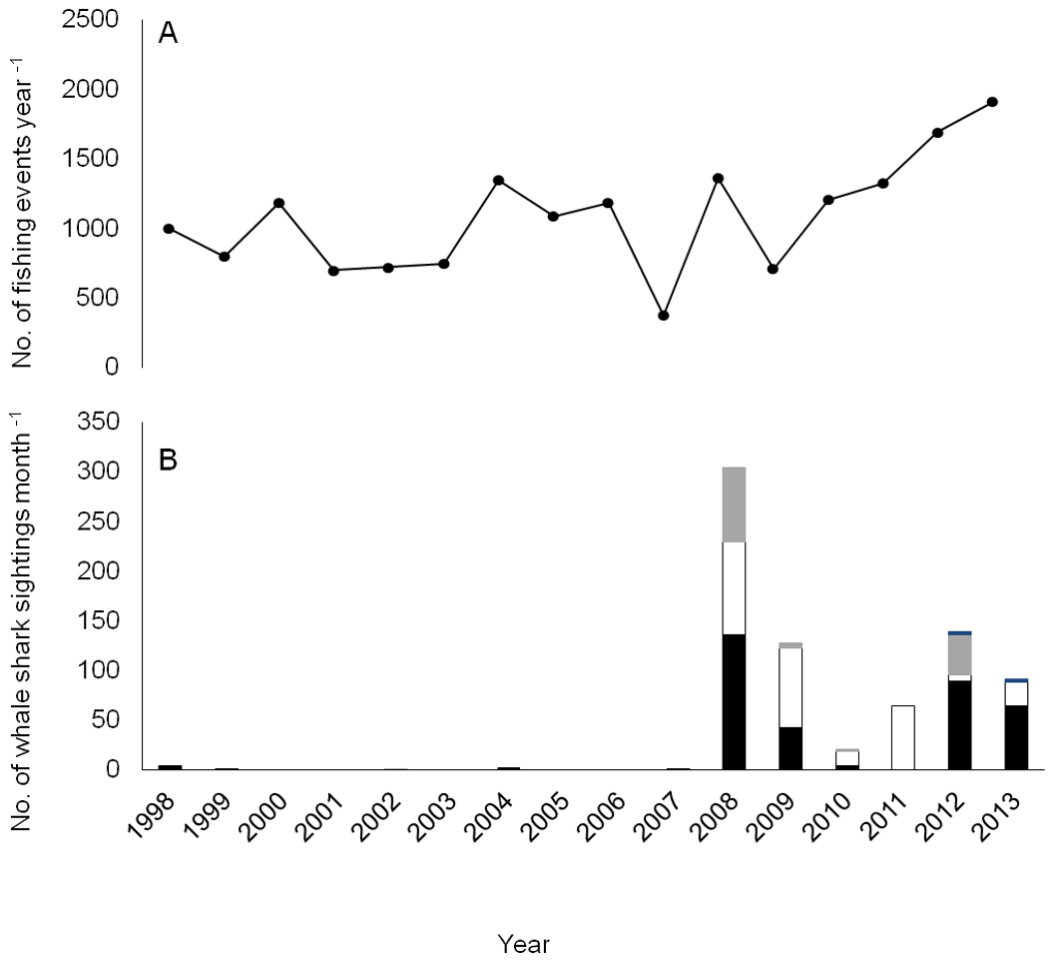
16 years of whale shark sightings in the Azores EEZ. (A) The annual cumulative effort of the pole and line tuna fishery expressed as the number of recorded events year^−1^. (B) The number of sightings across years for each of the month within whale shark season: April-June (dark grey), July (black), August (white) and September (light grey).

There were a total of 17339 fishing events between 1998 and 2013, of which 1443 (12.1%) had an associated whale shark sighting and resulted in a successful fishing event, i.e. the catch of tuna. Only on six occasions was there a sighting of a whale shark that did not result in a successful fishing event. The distribution of tuna fishing effort around the archipelago was predominantly focused in areas relatively close to the islands (Figure 1A). Effort also varied annually between 433 fishing events in 2007 and 1854 events in 2013 (Figure 2A) The filtering of the fishing events using the SU approach (monthly presences SU^−1^) resulted in a total of 758 monthly whale shark presences.

The most striking result is the dramatic increase of whale shark sightings in 2008, with 302 monthly sightings in that year alone (Figure 2B). Furthermore, annual sightings across the season had been null or very rare prior to 2008 (maximum four sightings in 1998) but thereafter sightings fluctuated between 21 in 2010 and 140 in 2012, always substantially higher in contrast with the pre 2008 period. Since 2008, there has been a certain degree of variation in sightings between months. Peak sightings shifted from an early peak in July 2008 to a later peak in August 2011 before returning to an earlier peak again in 2012 and 2013. The initial sightings were also made much earlier in the season during April and May, although quite rare.

**Figure 3:**
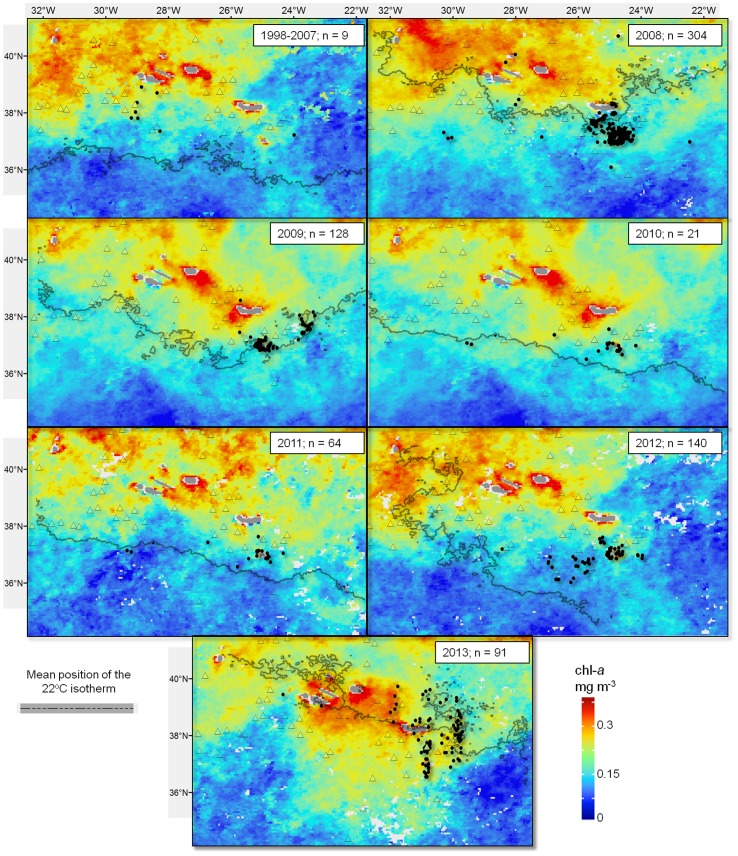
Geographic variability of whale shark sightings versus the local oceanography across the Azores EEZ between 1998 and 2013. Each panel shows the annual positions of each monthly sighting (black dots), the average chl-a biomass and the average location of the 22°C isotherm for the whale shark season (July to September). The few pre-2008 sightings are grouped in a single panel together with the average 22°C average isotherm for that whole period. Location of larger seamounts is also shown (triangles).

Sightings of whale shark were concentrated around the eastern group of islands, with the exception of 2012 (Figure 3). These sightings were mostly located around the southernmost island of Santa Maria, but a certain degree of spatial variation existed within this pattern, with clusters around the south coast of the neighbouring island of São Miguel or to the east – southeast of the archipelago. In contrast, 2012 showed the greatest deviation in the spatial pattern of whale shark sightings, as these were concentrated around the large Princess Alice Bank located to the SW of Faial and, for the first time, also made off the western islands. In 2013 the main distribution of sightings returned to the eastern islands, although now more dispersed along a North - South transect.

**Figure 4:**
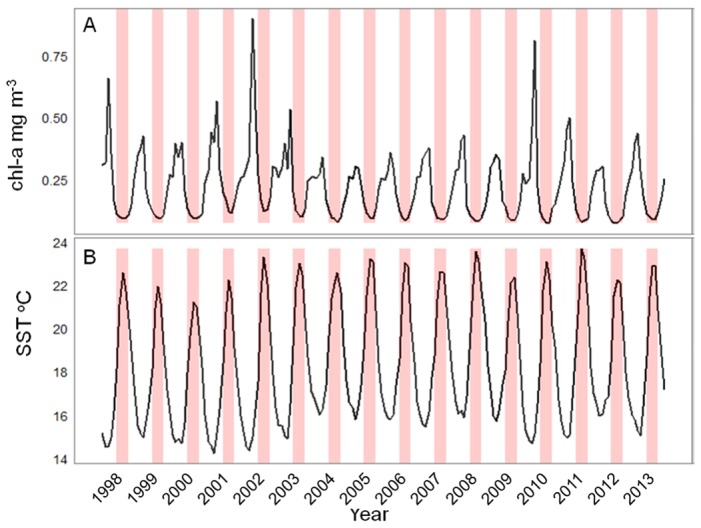
The primary productivity and thermic temporal signatures in the Azores EEZ. Time series of (A) chl-a biomass and (B) SST across the whole study period averaged for the entire study area. Highlighted periods (in red) indicate the time of year when peak whale shark sightings occur (July-September).

**Figure 5:**
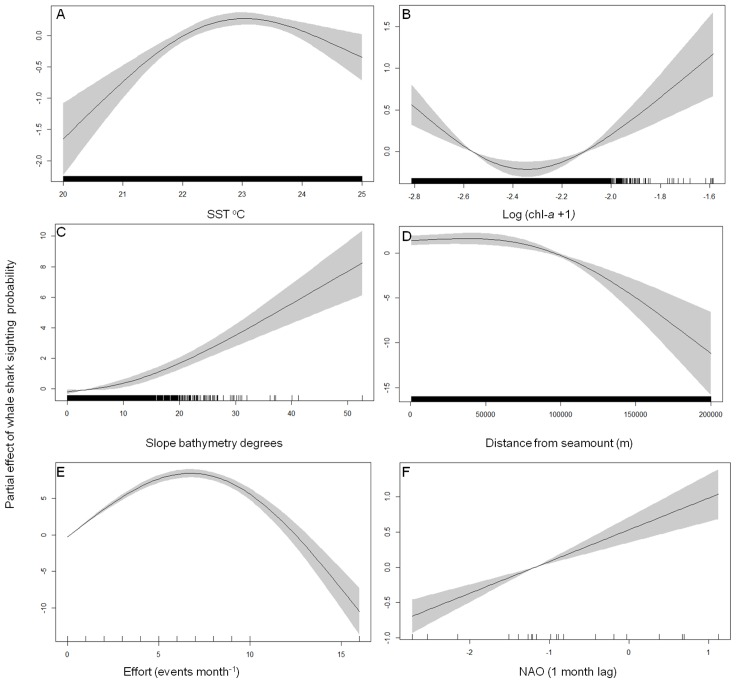
The influence of environmental variables on whale shark sightings. The partial residual plots for the five significant variables retained in the final GAM model, showing the probability of detecting a whale shark (black line  =  mean; shaded grey  = 95% CI) with changing environmental and spatial characteristics: (A) sea surface temperature, (B) primary productivity, (C) the slope of the underlying bathymetry, (D) distance to nearest seamount, (E) tuna fishing effort and (F) the North Atlantic Oscillation index.

**Figure 6:**
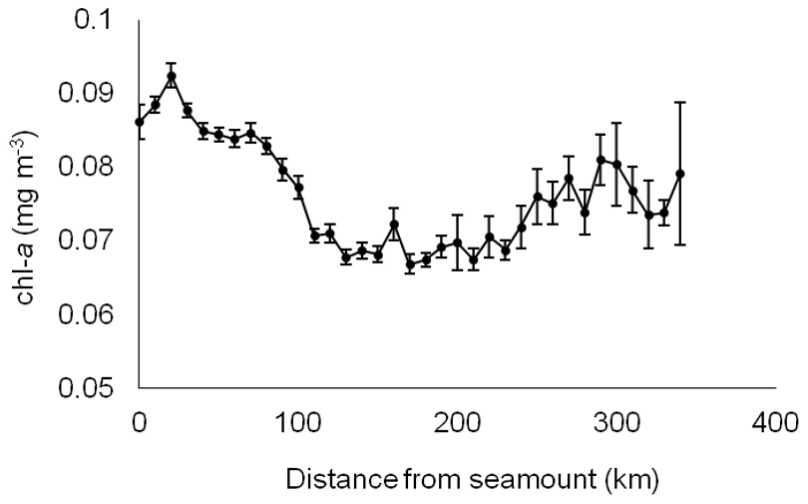
Increased productivity closer to seamounts. The average chlorophyll biomass (mg m-3 +/− SE) derived from satellite sensed chl-a binned into 10 km categories according to the distance of each pixel from the nearest seamount summit.

The full interannual time series of average SST and Chl-a for the study area show that sightings took place during the summer months when SST was at its highest while chl-a started to decrease after the spring bloom but also that there was a reasonable amount of interannual variability in both measures (Figure 4). Peak temperatures occurred in 2008 and 2011 with 2011 recording peak temperatures that were ∼0.5°C warmer than all years prior to 2008. Whale shark sightings took place associated to an overall SST envelope between 22 and 24°C. The broadscale temperature gradients also varied from year to year and, most importantly, appear to have followed an interannual trend similar to that of whale shark sightings. Location of the 22° isotherm varied in latitudinal position across the years but the orientation has also changed, particularly in 2012 and 2013. During pre-2008 years, the average position of the 22^o^ isotherm during the peak season (July - September) was south of the islands following an east-west orientation. In 2008, when whale shark sightings peaked, the isotherm was found much further north before returning to a more southerly position during 2009 and 2010. From 2011 onward the temperature gradients have seen a change moving from an east-west orientation towards a more north west - south east orientation, noticeably in 2012 when the waters around the western islands were warmer than those in the central and eastern groups (Figure 3). Whale shark sightings generally followed this pattern. While chlorophyll-a showed a consistent interannual broad scale pattern of an increasing south-north biomass gradient, there was a high degree of variation along the same latitudinal band, especially at the local scale. The final GAM model retained five of the original eight predictor variables and explained 66.6% of the total variance (Table 2). The ROC was also strong with a value of 0.942 which implies that the model discriminates between presences and absences effectively. The AIC for the final model was 1941 compared with 2276 for the model that contained all variables. The variables that were retained were SST, chl-*a*, distance from the seamount, slope and fishing effort. The most significant factor was fishing effort, which accounted for approximately half of the explained variance (33.5%). The four environmental factors explained from 12.1% to 4.2% amount of the variance (Table 2). The trends observed in the partial effects plots (Figure 5) show that whale shark sightings in the Azores were associated with several spatial and spatio-temporal conditions as well as changes in the fishing effort. Whale shark sightings tended to occur most often when the oceanic conditions of SST were 22^o^ (Figure 5A) and in areas of enhanced chl-*a* biomass (Figure 5B). Spatially, whale sharks were found to be closely aligned with the seamounts (Figure 5C) and in areas where the mean slope of the underlying bathymetry is larger (Figure 5D). An increase in effort from 0 to 3 events month^−1^ clearly raised the probability of detecting a whale shark in a particular SU but further increases in effort did not change that probability significantly (Figure 5E). Finally, there was a clear, non-autocorrelated increase of chl-*a* biomass within the vicinity of seamounts at about the same distance from the seamount summit that whale shark sightings peaked (Figure 6).

**Table 2:** Model results for whale shark detections.

Predictors	Resolution	
	*Deviance explained (%)*	*Chi sq.*
*Fishing effort*	28	912
*chl-a*	11.1	124.6
*Slope*	10.5	97.35
*SST*	4.2	35.48
*NAO*	2.0	34.53
*Distance to seamount*	1.8	31.04
*Distance to Shore*	-	-
*Depth*	-	-
***Total***	57.6	

The amount of deviance explained by each of the five predictor variables in the final GAM model and associated the chi squared (Chi sq.) value. All predictors were significant at a threshold of p<0.001.

## Discussion

### Regional patterns, migrations and the role of fringe habitats

Over the 16 year period of this study we found that the probability of occurrence of the whale shark in the wider Azores region increased drastically in 2008. Prior to this, and for a full decade, these large animals had only been sighted sporadically in spite of the large fishing effort across the Azorean EEZ. Thereafter, they became frequent summer visitors to the region. In addition, we found that SST helps predicting the probability of whale shark occurrence in the region across the years, providing a plausible explanation for the post 2007 shift at the regional scale.

As with most fisheries datasets, the survey effort in this study was not uniformly distributed, which can be problematic when discerning the effects of environmental cues on the patterns of the fishery-dependent occurrences. The pole-and-line tuna fishery in the Azores operates all over the EEZ but the vessels will often shift between areas where higher catches are expected based on recent catches (i.e., within the last couple of weeks). However, we believe that this variation did not significantly impact sightings of whale sharks because there is a very strong association of whale sharks with tuna in the region, as ascertained by the fact that nearly all sightings coincided with a successful fishing event. In addition, the probability of whale shark occurrences within a given 4 km×4 km SU did not increase with a concomitant increase in fishing effort above a small threshold (3 events SU^−1^). It should be noted that the highest distribution of fishing effort was concentrated around the central islands, however sightings in this area were rare, with only 10 sightings made within 20 km of the coastlines of the central islands. If whale sharks were present anywhere in the region in substantial numbers prior to 2008 one could expect larger whale shark associated catches and, concomitantly, higher whale shark sightings. Therefore, the inter-annual variation found in sightings should largely reflect a true variation in whale shark visits to the region.

This spatiotemporal pattern of regional occurrence linked to the thermic regime at Azorean latitudes in the summer must be put into context with the highly seasonal migratory behaviour of the species [5], [8]. If whale sharks occurring in the Azores during the summer come from any of the North Atlantic tropical areas where they are known to occur or aggregate annually, such as the Caribbean/Gulf of Mexico and Gulf of Guinea/West Africa [16], [26]–[27] (Figure 1), it would presumably take them some time to reach the higher latitudes of the Azores, as those areas are in the region of 2,500 to 6,000 km away from the region. Whale sharks are able to cover distances larger than this across the open ocean [9]–[10] but they possibly do so at considerable energetic costs. Additionally, the summer peak SST in the Azores region is always below the narrow optimal thermic envelope for whale shark (<26.5°C) described for other oceans [11], [16]. Therefore, periodic migrations up north and the concomitant expansion of whale shark oceanic habitat to the Azores might be worthwhile only if migrating individuals can access important local resources (e.g. food, access to mates) while keeping within temperatures warm enough to reduce the metabolic costs of migration [11]. This would explain the clear association seen between the annual occurrences and the 22°C temperature threshold. Such a constraining environmental effect is further noticeable when comparing the match between the geographic location and shape of the 22°C isotherm and the whale shark occurrences across the EEZ within a given season. In particular, there was a striking match between the change from a south-north to a west-east orientation of the isotherm in 2012 and the geographic dislocation of whale shark sightings from the southeast to the south central and western areas of the archipelago in that year. The fact that the interannual change in orientation of the SST gradients in the region appears to be a common mesoscale oceanographic feature [28] clearly indicates that, once whale sharks reach the region, their patterns of pelagic habitat use also responds to these features.

This relationship and the sharp post-2007 increase highlight the possibility that broadscale shifts in the regional biological and physical dynamics driven by climate change may be causing a shift in whale shark oceanic habitat use. In particular, it is most likely that we are witnessing a bottom-up response by the Atlantic whale shark population to changes in lower trophic level organisms (plankton, small fish), which have been evident in the pelagic ecosystem of the North Atlantic for some time [29]. Tropical Atlantic SSTs have shown a steady rise in recent years in accordance with trends found throughout the North Atlantic basin [30]. Changes in the SST, which represent the principal manifestation of climate change in our oceans, have been linked to large changes in the pelagic ecosystem, particularly in the North Atlantic [31]. Poleward extensions in the distribution of plankton [32] and fish species [4] of southern, more tropical affinities across Europe, as a result of the warming of coastal and oceanic waters, have been observed. This warming has led to a widening of the “tropical belt”, forcing an increase in stratification intensity and a decrease in the depth of the mixed layer, which has seen a ten-fold decrease in zooplankton biomass in the tropical ocean [33] and a four-fold decrease in regionally productive upwelling zones in the Gulf of Guinea [34]. This may be viewed as a trophic response to strong bottom-up regulation, as decreases in mean chl-*a* levels in tropical regions have been positively linked with a decrease in mixed-layer depth caused by continued ocean warming [35]–[36].

Finally, although there is no size information in our observer dataset, sightings of the whale shark appeared to be comprised almost entirely of adult (≥8 m) and large-sized (>10 m) individuals. This is supported by the high consistency of reports from tuna boat skippers (personal observation), local sharkwatching operators (Nuno Sá, personal communication; personal observations) and by our own observations in the field during three years of tagging operations around Santa Maria. If smaller, juvenile individuals (2–7 m) were frequent in the region, their occurrence would have been detected given their characteristic near-surface behaviour at their aggregation sites [10]. This pattern contrasts with the typical juvenile composition of the whale shark aggregations at tropical sites [8] and strengthens the hypothesis that the Azores region is part of the wider oceanic habitat in the North Atlantic for adult whale sharks, of which little is known globally [8]–[9], [14], [16].

### Local scale patterns: what brings whale sharks up north?

Within the region, there was a clear spatial pattern in whale shark sightings, namely the disproportionate amount of individuals appearing around the island of Santa Maria. This accumulation of sightings appears to reflect a true aggregation of individuals because our filtering procedure of monthly 4 km×4 km cell sightings is very conservative as to the possibility of repeated individual sightings which would require the animal to stay or repeatedly return to a cell in consecutive months.

To start with, the accumulation of sightings off Santa Maria should reflect the fact that average seasonal SST is indeed higher and typically above the position of the 22°C isotherm in that area as opposed to the other island groups. However, it also seems to be in agreement with the hypothesis that individuals must use cues other than large-scale oceanographic features (such as temperature) to orient their migrations and patterns of oceanic habitat use, at least once they reach the region. The NAO index is one of the dominant modes of climate variability in this region and captures much of the variability in climate-ocean interactions, including SST. In the tropical Atlantic, strong positive associations with the NAO have been detected in both phytoplankton [37] and zooplankton [38] populations. Positive NAO years shift the timing and abundance of the chl-*a* maximum further north and much later causing tropical blooms to be later and smaller in magnitude [39]. Whale shark occurrences in Ningaloo Reef, Australia have been found to be closely associated with the Southern Ocean Index [15]. There was a significant effect of the NAO _winter_ index as a change towards positive NAO phases increase the probability of whale shark appearances in the region. A longer time series would be necessary to verify this effect.

Access to food resources is one major possible cue. At the archipelago scale, there was a general increase in whale shark sightings after a given threshold in chl-*a* was reached, yet we found no evidence that the area around Santa Maria holds increased primary productivity In fact, there was a general increase in productivity around the other islands but not this one, which lies further south and therefore above what might be the optimal water temperature for that productivity to develop. The timing of whale sharks sightings in the area coincide with a period of highest SST and lowest chl-*a* values suggesting a potential lagged response between whale shark an chl-*a* as whale sharks actively feed on zooplankton and small planktivorous fish [5], [7] and, unlike the large grazer basking shark (*Cetorhinus maximus*), do not graze directly on algae [40]. Whale sharks have been seen and documented feeding on schools of juvenile horse mackerel (*Trachurus picturatus*) around Santa Maria (Nuno Sá, personal communication). These and other small planktivorous fish such as boarfish (*Capros aper*) and snipefish (*Macrorhamphosus scolopax*) are very abundant in the epipelagic layers over the island's slopes and nearby seamounts, and constitute main prey items of other pelagic predators that frequent these habitats in the region including seabirds [41], fishes [42] and sharks [43]. Our results also suggest that whale sharks may orient themselves with respect to seamounts and areas of steeper bottom slope within the area, as both productivity and sightings were indeed higher close to these topographic features. These patterns correlates well with previous findings for the region that report a peak in the biomass of marine top predators near but not over seamount summits, i.e., at their slopes, and support the hypothesis that this behaviour is adaptive by resulting in increased feeding opportunities in these areas [44]. Therefore, it is very well possible that the aggregation of whale sharks at particular areas once they reach the region reflects local abundance of prey, even if this behaviour is constrained at a first level by the larger scale environmental cues, such as temperature, that determine their migration probability.

Regardless of the whale shark feeding ecology in the region, it is worth noting that the Azorean aggregations could serve other functions such as increased opportunities for mature individuals to engage in social interaction, including mating. At present, there is no evidence to support or disproof this hypothesis, other than the anecdotal evidence that they are essentially composed of adult individuals. It is vital that we explore the demographic composition and the fine scale behaviour of the individuals in Azorean waters, given the profound lack of knowledge on the species' adult and mating grounds [8], [41].

### Conclusions

Based on a unique dataset spanning 16 years, we have demonstrated that changes in the mean annual summer SST are an important factor in strengthening the probability that whale sharks would migrate into the higher-latitude, oceanic habitat around the Azores, and that this region is at a thermal boundary for this species. As a result, the region has recently attracted seasonal numbers that highlights the significance of the Azores in the global network of well-established areas of seasonal aggregations in other regions [3], [8], [9], [12], [13]. Yet, the relevance of this region as oceanic habitat and its connectivity with other habitats across the Atlantic needs to be clarified. While we can reasonably assume that the recent influx of whale shark individuals into Azorean waters is related to the large-scale temporal changes in the tropical climate, the reasons for the spatial variability within the region and, in particular, the local aggregations remain unknown, although local productivity related to seamounts appears to play a major factor role.

Whale sharks have been actively hunted in some areas of the Indo-Pacific [45], inadvertently captured in tropical pelagic fisheries [46], [ 47], and suffer injuries from boat traffic in certain coastal aggregations [8], prompting growing conservation concerns and leading to the species' IUCN designation as “Vulnerable” [48]. Our findings underline the potential for an increase of the wider Azores region's importance as an oceanic habitat for the whale shark in the North Atlantic in years of exceptionally high water temperature, and for a concomitant shift in the whale shark distribution within the Atlantic Ocean, as predicted by global modelling studies [17]. In the future, such shifts need to be placed in the context of decadal and very long-term changes in this ocean. The use of telemetric studies, the continuation of the fisheries observer program and the establishment of a monitoring study directed at the aggregation areas within the region will be most useful to further address these questions in greater detail.
